# Facial pyoderma gangrenosum-like lesions in the setting of granulomatosis with polyangiitis

**DOI:** 10.1016/j.jdcr.2026.07.004

**Published:** 2026-07-07

**Authors:** Lina M. Ferrari, Scott A. Elman, Jose A. Jaller

**Affiliations:** Dr. Phillip Frost Department of Dermatology and Cutaneous Surgery, University of Miami Miller School of Medicine, Miami, Florida

**Keywords:** cutaneous vasculitis, facial lesions, granulomatosis with polyangiitis, neutrophilic dermatosis, PR3-ANCA, pyoderma gangrenosum-like lesions, systemic vasculitis

## Case description

**Fig 1:**
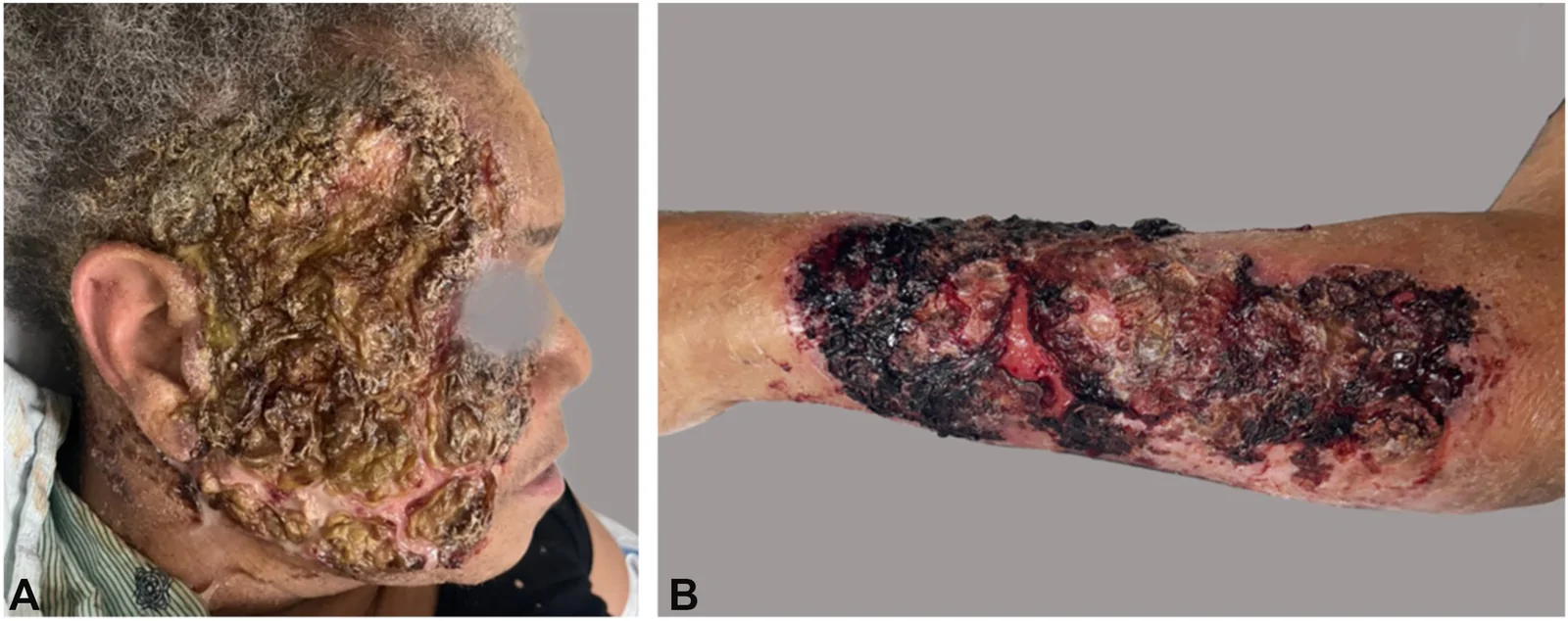
Verrucous, vegetating plaques with hemorrhagic crust in patient 1: **(A)** right cheek and **(b)** left forearm.

A 67-year-old woman presented with a history of painful longstanding, sharply demarcated, verrucous, vegetating plaques with hemorrhagic and serosanguinous crust on her bilateral cheeks, right preauricular region, left forearm, vulva, buttock, and abdomen (Fig 1, *A* and *B*). She reported her disease began in 2022 with a nodule on each cheek, one following incision and drainage of a cyst and another after an insect bite, that progressed into large hemorrhagic plaques. There was no history of prior iodinated contrast exposure. The following year she was hospitalized for extensive lesions and workup revealed positive cytoplasmic-ANCA (c-ANCA) and anti-proteinase 3 antibodies, with negative perinuclear-ANCA (p-ANCA), anti-myeloperoxidase (MPO) antibodies, antinuclear antibody, and fungal and mycobacterial studies. Histopathology demonstrated neutrophilic and granulomatous inflammation without vasculitis and computed tomography imaging of the sinuses demonstrated a large nasal septal defect with surrounding soft tissue thickening extending into the superior nasal cavity, erosive changes of the posterior hard palate, and extensive paranasal sinus opacification, findings consistent with granulomatosis with polyangiitis. The patient had facial plaques that persisted for 4 years and became progressively larger and painful. Prior treatment with intravenous Rituximab was unsuccessful.

**Question: Which finding most strongly supports granulomatosis with polyangiitis as the underlying diagnosis rather than idiopathic pyoderma gangrenosum?**

A. Pathergy following minor trauma
B. Negative bacterial and fungal cultures
C. Dense neutrophilic inflammation on histopathology
D. Positive anti proteinase 3 antibodies
E. Rapid response to systemic corticosteroids

Answer: D.

## Answer discussion

Granulomatosis with polyangiitis (GPA) is a systemic necrotizing vasculitis that classically affects small to medium sized blood vessels of the respiratory tract, kidneys, and skin and is most commonly associated with anti-proteinase 3 antineutrophil cytoplasmic antibodies.^1^, ^2^, ^3^ Ulcerative lesions with undermined, raised erythematous-violaceous edges mimicking pyoderma gangrenosum (PG) represent a rare cutaneous manifestation of systemic GPA and may be the initial or predominant presentation.^2^

Idiopathic PG is a neutrophilic inflammatory condition characterized by painful pustules or nodules, most often affecting the lower extremities, that evolve into ulcers with purulent drainage and violaceous, undermined borders.^4^ Facial involvement is uncommon and should prompt evaluation for underlying systemic disease. Histopathologic findings in PG are often nonspecific, typically demonstrating neutrophilic inflammatory infiltrate, and diagnosis is therefore made by exclusion of infection, neoplasia, and other inflammatory or vasculitic processes.^4^ Given this diagnostic overlap, recognition of systemic and serologic features becomes essential.

In this patient, positive proteinase 3-ANCA serology as well as sinonasal involvement provides strong diagnostic support for GPA and helps distinguish GPA-associated PG-like lesions from idiopathic PG. Recognition of this distinction is critical as timely diagnosis and treatment of the underlying systemic process are essential in optimizing outcomes and minimizing morbidity.

Management of disfiguring and painful ulcerative facial plaques in this setting requires treatment of underlying vasculitis, as control of systemic disease is necessary for cutaneous healing. PG-like lesions associated with GPA are often refractory to standard immunosuppression, however, rituximab, a monoclonal antibody directed against CD20, has shown efficacy in remission induction similar to that of cyclophosphamide.^5^

## Conflicts of interest

None disclosed.
